# Supplementary material for: From sequence to enzyme mechanism using multi-label machine learning
Source: BMC Bioinformatics. 2014 May 19;15:150. doi: 10.1186/1471-2105-15-150 (PMC4229970; doi:10.1186/1471-2105-15-150)
Supplement: Additional file 2 — Java code of ml2db. Additional file ml2db_code.tar.gz contains the Java source code to run the multi-label machine learning experiments and save the results to database. The code’s Javadoc is included. [file 1471-2105-15-150-S2.zip › additional file 2/ml2db/ml2db/doc/mulan/data/IterativeStratification.html]

IterativeStratification


JavaScript is disabled on your browser.


- Overview
- Package
- Class
- Use
- Tree
- Deprecated
- Index
- Help

- Prev Class
- Next Class

- Frames
- No Frames

- All Classes

- Summary:
- Nested |
- Field |
- Constr |
- Method

- Detail:
- Field |
- Constr |
- Method


mulan.data

## Class IterativeStratification

- java.lang.Object
- - mulan.data.IterativeStratification

- All Implemented Interfaces:
  :   Stratification, weka.core.TechnicalInformationHandler

  ---

    

  ```
  public class IterativeStratification
  extends java.lang.Object
  implements Stratification, weka.core.TechnicalInformationHandler
  ```

  Class for stratifying data based on the iterative stratification method

  Version:
  :   2012.05.08

  Author:
  :   Konstantinos Sechidis, Grigorios Tsoumakas

- - ### Constructor Summary

    Constructors

    Constructor and Description

    `IterativeStratification()`

    Default constructor

    `IterativeStratification(long seed)`

    Constructor setting a specific random seed
  - ### Method Summary

    Methods

    | Modifier and Type | Method and Description |
    | --- | --- |
    | `weka.core.TechnicalInformation` | `getTechnicalInformation()` Returns an instance of a TechnicalInformation object, containing detailed information about the technical background of this class, e.g., paper reference or book this class is based on. |
    | `MultiLabelInstances[]` | `stratify(MultiLabelInstances data, int folds)` Creates a number of folds via stratified sampling |

    - ### Methods inherited from class java.lang.Object

      `equals, getClass, hashCode, notify, notifyAll, toString, wait, wait, wait`

- - ### Constructor Detail


    - #### IterativeStratification

      ```
      public IterativeStratification()
      ```

      Default constructor


    - #### IterativeStratification

      ```
      public IterativeStratification(long seed)
      ```

      Constructor setting a specific random seed

      Parameters:
      :   `seed` -
  - ### Method Detail


    - #### getTechnicalInformation

      ```
      public weka.core.TechnicalInformation getTechnicalInformation()
      ```

      Returns an instance of a TechnicalInformation object, containing detailed
      information about the technical background of this class, e.g., paper
      reference or book this class is based on.

      **Specified by:**
      :   `getTechnicalInformation` in interface `weka.core.TechnicalInformationHandler`

      Returns:
      :   the technical information about this class


    - #### stratify

      ```
      public MultiLabelInstances[] stratify(MultiLabelInstances data,
                                   int folds)
      ```

      **Description copied from interface: `Stratification`**

      Creates a number of folds via stratified sampling

      **Specified by:**
      :   `stratify` in interface `Stratification`

      Parameters:
      :   `data` - a multi-label dataset
      :   `folds` - the number of folds to sample

      Returns:
      :   an array of multi-label datasets, one for each fold


- Overview
- Package
- Class
- Use
- Tree
- Deprecated
- Index
- Help

- Prev Class
- Next Class

- Frames
- No Frames

- All Classes

- Summary:
- Nested |
- Field |
- Constr |
- Method

- Detail:
- Field |
- Constr |
- Method
